# Utility of telephone survey methods in population-based health studies of older adults: an example from the Alberta Older Adult Health Behavior (ALERT) study

**DOI:** 10.1186/1471-2458-14-486

**Published:** 2014-05-22

**Authors:** Jeff K Vallance, Dean T Eurich, Paul A Gardiner, Lorian M Taylor, Gillian Stevens, Steven T Johnson

**Affiliations:** 1 Faculty of Health Disciplines, Athabasca University, Athabasca, AB, Canada; 2 School of Public Health, University of Alberta, Edmonton, AB, Canada; 3 Centre for Longitudinal and Life Course Research, School of Population Health, The University of Queensland, Brisbane, Australia; 4 Nutrition Services, Alberta Health Services, Edmonton, AB, Canada; 5 Department of Sociology, Population Research Laboratory, University of Alberta, Edmonton, AB, Canada

**Keywords:** Health behavior, Computer-assisted telephone interviews (CATI), Random digit dialing, Response rate, Older adults

## Abstract

**Background:**

Random digit dialing is often used in public health research initiatives to accrue and establish a study sample; however few studies have fully described the utility of this approach. The primary objective of this paper was to describe the implementation and utility of using random digit dialing and Computer Assisted Telephone Interviewing (CATI) for sampling, recruitment and data collection in a large population-based study of older adults [Alberta Older Adult Health Behavior (ALERT) study].

**Methods:**

Using random digit dialing, older adults (> = 55 years) completed health behavior and outcome and demographic measures via CATI. After completing the CATI, participants were invited to receive a step pedometer and waist circumference tape measure via mail to gather objectively derived ambulatory activity and waist circumference assessments.

**Results:**

Overall, 36,000 telephone numbers were called of which 7,013 were deemed eligible for the study. Of those, 4,913 (70.1%) refused to participate in the study and 804 (11.4%) participants were not included due to a variety of call dispositions (e.g., difficult to reach, full quota for region). A total of 1,296 participants completed telephone interviews (18.5% of those eligible and 3.6% of all individuals approached). Overall, 22.8% of households did not have an age 55+ resident and 13.6% of individuals refused to participate, Average age was 66.5 years, and 43% were male. A total of 1,081 participants (83.4%) also submitted self-measured ambulatory activity (i.e., via step pedometer) and anthropometric data (i.e., waist circumference). With the exception of income (18.7%), the rate of missing data for demographics, health behaviors, and health measures was minimal (<1%).

**Conclusions:**

Older adults are willing to participate in telephone-based health surveys when randomly contacted. Researchers can use this information to evaluate the feasibility and the logistics of planned studies using a similar population and study design.

## Background

In Canada, few efforts have been established to monitor and track the health behaviors (and related health outcomes) of older adults. Older adults have some of the poorest health behavior indices which are often associated with an increased risk of chronic disease [1,2]. Monitoring these behaviors, and gaining a deeper understanding of the factors associated with various health behaviors in this population can: a) provide relevant information for stakeholders upon which evidence-based interventions and programs can be developed and implemented, and b) facilitate the health and well-being of older adults.

Researchers have utilized a variety of sampling/recruitment strategies including social media, health registry/database/chart review, print media, and public service announcements [3]. In the context of older adults, several studies have used random digit dialing and subsequent Computer-Assisted Telephone Interview (CATI) methods to establish a study sample. However, details regarding the recruitment procedures, outcomes (e.g., data completeness, reasons for refusal, number of call attempts) and call dispositions (e.g., average number of call attempts, reasons for refusal) are limited [4-7]. Providing these details (and challenges) related to characteristics of telephone recruitment using random digit dialing and CATI survey methods for study recruitment may assist other researchers in their planning and execution of older-adult health-related initiatives utilizing these methods.

We recently completed participant recruitment and data collection for the population-based Alberta Older Adult Health Behavior (ALERT) study. The primary objective of this paper was to describe the implementation and utility of using random digit dialing and CATI for sampling, recruitment and data collection in the ALERT study. Secondary objectives were to: a) examine the utility of using objective measures in telephone-based population studies, and b) describe follow-up completion and data completeness rates using these approaches.

## Methods

The ALERT study was a large population-based study of the health behaviours and objective outcomes of older adults (≥55 years of age) conducted using random digit dialing and CATI. All subject recruitment and data collection were conducted through a centralized research unit - the Population Research Laboratory (PRL) at the University of Alberta (Edmonton, Alberta, Canada). The PRL is a member of the Association of Academic Survey Research Organizations (AASRO) and assists academic researchers and policy makers in designing and implementing applied social science research projects. The PRL specializes in the gathering, analysis, and presentation of data about demographic, social and public issues and has particular expertise in administering CATI surveys. The PRL maintains a complement of highly skilled telephone interviewers and supervisors who are thoroughly trained in respondent selection procedures, questionnaire instructions, and neutral probing.

The ALERT Study was approved by the University of Alberta’s Health Research Ethics Board as well as the Athabasca University Research Ethics Board. Prior to initiating the survey portion of the telephone interview, participants were assured that their participation is voluntary and that their answers are confidential and stored in a secured database and used only for study purposes. Participants were also informed they had have the right to end the interview at any time.

### Random digit dialing

A random digit dialing approach was used to ensure that households in each health zone had an equal chance to be contacted whether or not their household was listed in a telephone directory. The sampling frame of telephone numbers updated to 2012 was based on landlines and excluded businesses, unlisted cellular phone exchanges, and government exchanges. The typical structure of a telephone number in the province of Alberta (and across Canada) is: Area code (NPA)-Prefix/Exchange (NXX)- Phone Number (XXXX). Since 1986, the PRL has maintained a set of eight digit working banks (NPA-NAA-XX) for the province of Alberta. The PRL works with a vendor to update the list regularly to add in new area codes (3 digits), new exchanges (3 digits assigned to certain geographies in the province) and observed densities of residential and business telephone numbers assigned at the two-digit level. The last two digits in the phone number are then randomly seeded and generated in the range 00–99 and appended with replacement to the working bank file based on the number of telephone numbers projected to reach the sample size desired. Within the 10-digit numbers generated the PRL purges the duplicate phone numbers. However, even at the two-digit level of generation, a substantial number of not-in service numbers and business numbers are still generated. The sample of numbers is sent out to a vendor for pre-dialing (screening) to filter out these numbers before data collection projects that require interviewer dialing. The main strength of random digit dialing is the ability to sample all telephone households in the province, not simply households on a particular directory or commercial list [8].

### Measures

Data collection consisted of two stages. In stage I, data were collected via CATI. *Physical activity* was assessed using the leisure score index (LSI) of the Godin Leisure-Time Exercise Questionnaire (GLTEQ) [9]; *Sitting* (*i.e*., *sedentary behavior*) was assessed using a newly developed tool designed to assess total past year sitting behaviors and time in the following domains; sleeping and napping, transportation, work and volunteering, and light leisure and relaxing. It was designed for use in epidemiologic studies, and can be administered by interviewer of by written questionnaire. [10]; *Health*-*related quality of life* was measured using the four-week version of the RAND-12 Health Status Inventory (RAND-12) [11]; *Depressive symptoms* were assessed using the validated Patient Health Questionnaire-9 (PHQ-9) [12]; *Health literacy* was assessed using three health literacy screening questions developed and validated by Chew and colleagues [13]; *Medication adherence* was assessed using the Moriskey 8-item Medication Adherence Questionnaire [14]. *Chronic health conditions*, *demographic information* (e.g., age, income, education, employment), and *health information* (e.g., anthropometrics, smoking status) were assessed via self-report.

After confirming eligibility, and prior to the start of the interview, participants were asked if they were willing to wear a step pedometer for three days, and measure their waist size. In stage II of data collection, the PRL mailed a step pedometer (StepsCount SC-01, Deep River, Ontario, Canada), and a 60” waist circumference tape measure (Almedic, Stevens, Ontario, Canada) to each participant who consented and completed the survey interview, and agreed to receive the pedometer and tape measure. Participants were asked to start tracking their daily steps over a three-day period the following morning after receiving the package in the mail. Participants were encouraged to wear the pedometer on one weekend day. The package included detailed instructions on how to use the pedometer, how to record the number of steps for each day on a log sheet, and how to measure their waist circumference. To obtain the most precise waist measurement, participants were asked to complete the measure three times and perform all three measures at one time. After one week of sending the pedometer, the interviewers contacted the participants and recorded their three-day step count total and waist circumference. Participants were also able to e-mail their data to the project coordinator at the PRL. When there was no option to leave a message, the PRL sent a letter to the participants reminding them to submit their step count and waist circumference information.

### Pretesting

Prior to data collection, interviewers received training from the study principal investigator (JV) on survey content and administration of the survey procedures. Pre-testing commenced on a sample of 20 respondents and was initiated via random digit dialing to test the adequacy of the survey instrument including routing patterns, question wording and flow, adequacy of transitional statements, and to estimate the length of the interviews. In addition, the PRL assessed the mail out process and follow-up to pretest participants to obtain step counts and waist measurement information.

### Sampling design

The target population consisted of people who at the time of the survey were living in Alberta and could be contacted by direct phone dialing. Individuals were eligible to participate if they were at least 55 years of age, were able to walk unassisted (without the aid of a wheelchair or walking aid), community dwelling (not living in an aged care setting) and were able to complete a computer-assisted telephone survey in English. Sampling by gender, equally weighted across the zones based on estimates of population maintained by the province of Alberta, was conducted.

### Data collection

The PRL collected the main data using a CATI system. The PRL research coordinator formatted the ALERT instrument electronically and loaded a sample database of randomly generated telephone numbers onto the CATI system, which allocates telephone numbers to the interviewing stations after which the interviewer dials the number. To eliminate effects of seasonality on health and health behaviors (i.e., extreme hot and cold), the interviewing began on September 28, 2012 and was completed on November 20, 2012. This sampling time frame was chosen as all weather patterns could likely be experienced within these months in Alberta (e.g., ranging from warm summer heat in late September to potential snowfall in late November). All data collection was conducted from the centralized CATI facilities of the PRL at the University of Alberta. To obtain a greater response rate, telephone numbers allocated by the CATI system were dialed at least eight times or until a final disposition (such as a completed interview or a final refusal) was obtained. Telephone numbers were called on various days over a one-week period and at different times of day. After making contact, interviewers identified themselves, verified the telephone number, and then asked the screening questions for selecting the respondent. Before administering the questionnaire, the interviewer reviewed consent and confidentiality with the participants.

The PRL team took steps to ensure high quality data including initial extensive training, regular reporting and communication with the principal investigator (JV) on issues and concerns, periodic information updates and refresher trainings for the interviewers, continuous review of the data for missing values and inconsistencies, checking call dispositions, and conducting back-up interviewing by supervisors when required. Data were accumulated on a regular basis using the features of the CATI system and then imported into the IBM SPSS for Windows (version 20.0). The data were checked regularly. The data cleaning process included wild code, discrepant value, and consistency checks. Each variable was reviewed and evaluated. Missing data was defined as any data point where the participant indicated either 1) no response, or 2) don’t know/not sure.

## Results

The PRL made 102,977 dialings on 36,000 unique telephone numbers to obtain 1,296 completed interviews for the study (yield 3.6% per unique telephone number). Participants included in the study were proportionally representative of the five provincial heath zones sampled. On average, each completed interview took two call attempts to complete. Overall, 37% of call records were made on the first call attempt with approximately 18% of call records made on the second attempt (Table 1). Approximately 99% of all completed interviews were obtained by the sixth attempt. The median survey length for all respondents was 24.0 minutes and the mean survey length was 25.5 minutes (SD = 7.4). Overall, we enrolled 96% of respondents with 85% of calls and 88% with only 72% of calls.

**Table 1:** Number of call attempts for completed interviews and for all call records for main study

**Number of attempts**	**Completed interviews**			**All call records**		
	**Frequency(N)**	**Percent (%)**	**Cumulative percent**	**Frequency(N)**	**Percent (%)**	**Cumulative percent**
1	426	32.9	32.9	13,313	37.0	37.0
2	355	27.4	60.3	6,460	17.9	64.9
3	204	15.7	76.0	4,076	11.3	66.2
4	150	11.6	87.6	2,205	6.1	72.4
5	104	8.0	95.6	4,524	12.6	84.9
6	46	3.5	99.2	4,997	13.9	98.8
7	7	0.5	99.7	306	0.9	99.7
8+	4	0.3	100.0	119	0.3	100.0
Total	1,296	100	100	36,000	100	100

The most common final call disposition was ineligibility due to age (i.e., no residents 55 years of age or older in the household) (n = 8,193; 23%) (Table 2). Other reasons for ineligibility included business/fax line (n = 6,240), no answer/answering machine (n = 8,992), and number not in service (n = 3,587). Of the 7,013 individuals contacted and deemed eligible, 4,913 (70.1%) refused to participate in the study. Despite the option to only complete the telephone survey portion of the study, 290 individuals refused participation as they did not want to wear the pedometer. Further, there were 222 households in which the potential respondents were difficult to contact (e.g., away for the duration of the survey, unable to participate due to illness, or unwilling to participate due to illness or death in the family). Further, 182 were not eligible due to a full quota (i.e., region), 79 were reached via cellular phone and did not want to be disturbed, 19 claimed to be on the National Do Not Call List which does not apply to health research, and 12 indicated they would call back but never did. Thus, of the eligible households (n = 7,013), the overall participation rate was 18.5% (1,296 participants). One participant per household participated as spouses or partners were not eligible. Of the 1,296 participants, 75% (n = 967) agreed to be contacted about future health research studies by the research team.

**Table 2:** Final call dispositions for main study

**Final disposition number**	**Final outcome of call attempts**	**Total participants**	
		**n**	**%**
	**Completed interviews**	**1,296**	**3.6**
01	No answer	3,829	10.6
02	Busy	433	1.2
03	Answering machine	5,163	14.3
04	Line trouble	659	1.8
05	Quota full^1^	182	0.5
06	Call back^2^	343	1.0
07	Language problem	220	0.6
08	Not in service	3,587	10.0
09	Business, fax	6,240	17.3
10	Permanent no contact^3^	222	0.6
11	Claimed to be on the National Do Not Call List^4^	19	0.1
12	Ineligible - No 55+ in household	8,193	22.8
13	Ineligible – Unable to walk unassisted	214	0.6
14	Will call lab	12	0.0
15	Cell phone, do not want to be disturbed	79	0.2
16	8 or more call attempts	106	0.3
17	Refused	4,913	13.6
18	Refused pedometer	290	0.8
Total telephone allocated		36,000	100
Participation rate (%)		19.3	

^1^Female quota reached.

^2^Data collection already completed. Household not re-contacted.

^3^Households not available for the duration of the survey (e.g., on vacation, family crisis).

^4^Launched in September 2008 to legislate unsolicited marketing or sales calls.

For the 1,296 participants competing the CATI survey, the average age was 66.5 years (SD = 8.2), 555 (43%) were male, 1,221 (94%) were Caucasian and approximately half were retired (52%) (Table 3). The majority of participants were married (66%), had some level of advanced education (college or university; 62%), reported an annual income (individual) over $60,000 per year (55%), and had Internet access (86%). Statistically significant differences (using chi square tests) between men and women were observed for marital status, education, income, employment status, and some comorbidities (i.e., asthma, fibromyalgia, osteoarthritis, heart disease, thyroid condition, and mood disorder). All demographic (e.g., age, education), health behavior (e.g., physical activity, sedentary behavior, medication adherence), and health measures (e.g., HRQoL, depression) had <1% missing data, with the exception of income (18.7%). The primary source of missing data was item nonresponse, where the participant; a) did not provide an acceptable response to the survey item due to either refusal to answer or b) reported they did not know the answer to the item [15].

**Table 3:** Demographic details of study participants

	**Overall**	**Men N = 555**	**Women N = 741**	**P value**	**Missing data**
Age	66.5 ± 8.2	66.2 ± 8.1	66.6 ± 8.3	0.39	10
BMI	27.2 ± 5.1	27.2 ± 4.2	27.1 ± 5.7	0.69	17
**Marital Status**					
Single	73 (6)	32 (6)	41 (6)	<0.001	6
Married	846 (66)	409 (74)	437 (59)		
Common Law	41 (3)	19 (3)	22 (3)		
Divorced	123 (10)	38 (7)	85 (12)		
Separated	30 (2)	18 (3)	12 (2)		
Widowed	177 (14)	28 (7)	139 (19)		
**Education**					
Some high School	143 (11)	62 (11)	81 (11)	<0.001	1
High School	289 (22)	110 (20)	179 (24)		
Some College	231 (18)	79 (14)	152 (21)		
College/Univ Degree	565 (44)	252 (45)	313 (42)		
Trade	67 (5)	52 (9)	15 (2)		
**Income**					
<20 k	79 (8)	28 (6)	51 (9)	<0.001	242
20-39,999	226 (21)	69 (14)	157 (27)		
40-59,999	173 (16)	74 (15)	99 (17)		
60-79,999	161 (15)	76 (16)	85 (15)		
80-99,999	122 (12)	65 (14)	57 (10)		
>100 K	293 (28)	167 (35)	126 (22)		
**Employment status**					3
Full-time	361 (28)	191 (35)	170 (23)	<0.001	
Part-Time	140 (11)	49 (9)	91 (12)		
Unemployed, looking	24 (2)	8 (2)	16 (2)		
Not working, not looking	13 (1)	2 (0)	11 (1)		
Retired	678 (52)	275 (50)	403 (54)		
Homemaker	29 (2)	0 (0)	29 (4)		
Disability	22 (2)	11 (2)	11 (1)		
Retired but working	10 (1)	8 (1)	2 (0)		
Seasonal work	2 (0)	1 (0)	1 (0)		
Self-employed	14 (1)	8 (1)	6 (1)		
**Racial background**					
Caucasian	1221 (94)	516 (93)	705 (95)	0.14	3
Aboriginal	16 (1)	6 (1)	10 (1)		
Other	56 (4)	31 (6)	25 (4)		
**Comorbidities**					
Cancer	239 (18)	106 (19)	133 (18)	0.613	0
Diabetes	156 (12)	82 (15)	74 (10)	0.012	
Asthma	101 (8)	30 (5)	71 (10)	0.006	
Fibromyalgia	47 (4)	4 (1)	43 (6)	<0.001	
Osteoarthritis	345 (27)	86 (16)	259 (35)	<0.001	
Rheumatoid arthritis	114 (9)	48 (9)	66 (9)	0.921	
COPD	66 (5)	32 (6)	34 (5)	0.372	
Heart disease	146 (11)	79 (14)	67 (9)	0.004	
Stroke	33 (3)	16 (3)	17 (2)	0.594	
Bowel Disorder	95 (7)	32 (6)	63 (9)	0.067	
Thyroid condition	196 (15)	33 (6)	163 (22)	<0.001	
Kidney dysfunction	38 (3)	21 (4)	17 (2)	0.135	
Mood disorder	114 (9)	31 (6)	83 (11)	<0.001	
Internet access	1118 (86)	493 (89)	625 (84)	0.02	0

Numbers may not equal 1,296 due to missing data.

Data are presented as the mean (SD) for continuous variables (i.e., age, weight, and body mass index) and frequency (%) for categorical variables.

Demographic and clinical characteristics of participants were compared using analysis of variance and chi square tests where appropriate.

Of the 1,296 participants who completed the CATI survey, only 19 (1%) of people declined the pedometer and waist circumference follow-up for various reasons (e.g., going on extended vacation, emergency surgery). Eighty-five percent (n = 1,081) of the 1,277 participants who received the pedometer and waist circumference tape measure submitted their data to the PRL. Overall, 100% of participants provided step data on all three days and three sequential waist circumference measurements (i.e., there was no missing data). Of the sample, 625 participants voluntarily submitted this data via e-mail. The remaining 454 participants provided their data when they received a follow-up phone call from the interviewer. Total cost to complete the study (e.g., staff, pre test, data collection, postage, follow-up, supplies) was $115.90 (CDN) per participant.

## Discussion

Our study is one of the first to fully report on the implementation and outcomes associated with CATI survey with follow-up objectively measured data. We found that among older adults, random digit dialing is a feasible recruitment strategy in obtaining a population-based sample of older adults. Although the overall yield per telephone call is relatively low (3.6%), participation rates were relatively high among eligible individuals despite the inclusion of objectively measured data (19.3%). Indeed, of the final sample, 85% of participants (n = 1,081) completed and returned the objective assessments (step pedometer and waist circumference). Of these participants, data quality was excellent as 100% of participants provided step data on all three days and three waist circumference measurements.

Our participation rate of 19.3% among eligible individual is higher than previous reports where the average random digit dialing telephone survey response rates in the U.S. was approximately 9% (out of 761 surveys) [16]. While we were able to achieve a high participation rate, the general decline in participation rates for random digit dial telephone surveys has been well documented [17,18]. Refusing participation can be explained by a range of factors including time constraints (decreasing volunteerism), a general cynicism about research, the research may present as a burden, or disinterest in the survey topic [19]. Moreover, households are increasingly subject to telemarketing solicitation and automated telephone calls (i.e., ‘robocalls’), which may reduce people’s willingness to participate in legitimate surveys. However, in our study of older adults, call dispositions did not indicate an overwhelming sense of cynicism, burden, or lack of interest in the topic. Indeed, our data cannot take into account individuals screening or blocking unknown numbers using telephone technologies such as caller ID or call blocking (or a general refusal to answer the phone). While a high participation rate may lead to a higher degree of validity in the resulting data, research has demonstrated that low participation rates do not necessarily compromise the validity of the data [20].

Our data also suggests that the majority of participants in the study were contacted after the second attempt and virtually no benefits in recruitment were gained after 6 attempts. However, it should be noted that the overall yield per call is relatively low at 3.6%. We enrolled 96% of respondents with 85% of calls and 88% with only 72% of calls. Future researchers would experience cost savings by potentially stopping recruitment early. Although our data suggest that random digit dialing is highly feasible in relatively large populations, the utility of this approach in smaller and more restricted populations would be questionable. This has important implications for researchers who are conducting population-based studies where a large number of restrictions are included or where highly select and specific participants are required. Although one would have anticipated that inclusion of objective measures would have significantly hindered patient enrollment, our data suggest that older adults are willing and able to complete objective assessments beyond the telephone survey context. It is possible that participants perceived the step pedometer and waist circumference tape measure as an incentive to participating. Best practice guidelines for improving respondent cooperation for telephone surveys suggest that issuing incentives (monetory or non-monetary) has a strong impact on telephone survey participation rates [21,22]. Regardless, the use of health behavior assessment tools that have consumer appeal and usability appear to provide a mutual benefit for both the researcher and the participant.

Despite the strengths of our study including random digit dialing procedures, the use of trained interviewers, a geographically representative sample of older adults, and the use of objective physical activity ambulatory behavior (pedometers) and health assessments (waist circumference), there are several limitations that deserve mention. First, there is a growing trend towards the use of mobile phones. The generalizability of our sample, and studies that use similar methods, may become more limited as these individuals (who have no landline) are not represented in this survey design. As a result, researchers will need to develop additional techniques to reach these individuals. Second, the data provided may be biased towards socially acceptable response as opposed to the true feelings of the individual. There is no information regarding study non-responders to compare with study participants. However, using Statistics Canada 2012 census data we compared our sample to the general population on education and employment status. Our sample was representative of the population based on these variables as census data indicated 50.8% of Albertans over the age of 55 years had completed college or university [23], 44.9% were currently employed part or full time [24], and 59% were classified as overweight/obese (compared to 63.6% in our sample) [25]. We were unable to compare the samples on income as our study asked participants for combined household income as opposed to census data that collects individual respondent income. Selection bias may be occurring where only those individuals who were interested in health may have participated in the study.

## Conclusions

Our study suggests that older adults are interested in and willing to participate in telephone-based health surveys using random digit dialing and CATI. Further, our study suggests that these methods can be expanded to include not only traditional telephone-based collection of information but also objective measures of health behaviors and anthropometrics including walking and waist circumference via post. The majority of completed interviews (60.3%) were achieved on the first two call attempts. Researchers utilizing similar approaches to data collection may consider making only two call attempts for possible cost savings given the diminished benefits of making >2 call attempts. Based on comparison to national census data, older adults that participated in our study were fairly representative of the general population of older adults living in the province of Alberta. Researchers seeking to conduct studies of a similar design with older adults can expect an ~19% response rate of those eligible and ~4% of all random numbers called.

Random digit dialing and CATI are indeed common data collection tools in market research. However, with respect to the health context, random digit dialing and CATI use among samples of older adults is limited. Our manuscript provides readers with information on logistics and feasibility of recruiting older adults into similarly designed studies. Our data provides information in which readers can use to plan and budget for related research initiatives. Our study suggests that it is feasible to ask older adults to complete objective measures (i.e., pedometer steps and waist circumference) via post, after the telephone interview is complete. The older adults in our sample certainly demonstrated they were able to conduct these two assessments without any difficulties. Future research should continue to engage older adults in telephone-based health surveys with the addition of more objective and valid measures of health and health behaviour, such as the use of accelerometers to objectively measure physical activity and sedentary behavior.

## Competing interests

The authors declare that there are no competing interests.

## Authors’ contributions

JV conceived and coordinated the study, provided financial support/funding, and wrote the first manuscript draft. DE and ST were involved in the conception and coordination of the study, and provided critical feedback on multiple manuscript drafts. GS is the Director of the PRL and was involved in the design and conduct of the study. LT and PG provided assistance in the analysis of the data, and provided critical feedback on multiple manuscript drafts. All authors read and approved the final manuscript.

## Pre-publication history

The pre-publication history for this paper can be accessed here:

http://www.biomedcentral.com/1471-2458/14/486/prepub
